# Anti-Inflammatory Investigations of Extracts of *Zanthoxylum rhetsa*

**DOI:** 10.1155/2021/5512961

**Published:** 2021-03-06

**Authors:** Chureeporn Imphat, Pakakrong Thongdeeying, Arunporn Itharat, Sumalee Panthong, Sunita Makchuchit, Buncha Ooraikul, Neal M. Davies

**Affiliations:** ^1^Graduate School on Applied Thai Traditional Medicine Program, Faculty of Medicine, Thammasat University, Pathumthani 12120, Thailand; ^2^Department of Applied Thai Traditional Medicine, Faculty of Medicine, Thammasat University, Pathumthani 12120, Thailand; ^3^Center of Excellence in Applied Thai Traditional Medicine Research (CEATMR), Thammasat University, Pathumthani 12120, Thailand; ^4^Department of Agricultural Food and Nutritional Science, Faculty of Agricultural Life and Environmental Sciences, University of Alberta, Edmonton, AB T6G 2P5, Canada; ^5^Faculty of Pharmacy and Pharmaceutical Sciences, University of Alberta, Edmonton, AB T6G 2P5, Canada

## Abstract

*Zanthoxylum rhetsa* has been consumed in the diet in northern Thailand and also used as a medicament in ancient scripture for arthropathies. Thus, this study aimed to evaluate the activity of various extracts from differential parts of *Z. rhetsa* via inhibition of inflammatory mediators (NO, TNF-*α*, and PGE_2_) in RAW264.7 macrophages. The chemical composition in active extracts was also analyzed by GC/MS. The parts of this plant studied were whole fruits (F), pericarp (P), and seed (O). The methods of extraction included maceration in hexane, 95% ethanol and 50% ethanol, boiling in water, and water distillation. The results demonstrated that the hexane and 95% ethanolic extract from pericarp (PH and P95) and seed essential oil (SO) were the most active extracts. PH and P95 gave the highest inhibition of NO production with IC_50_ as 11.99 ± 1.66 *μ*g/ml and 15.33 ± 1.05 *μ*g/ml, respectively, and they also showed the highest anti-inflammatory effect on TNF-*α* with IC_50_ as 36.08 ± 0.55 *μ*g/ml and 34.90 ± 2.58 *μ*g/ml, respectively. PH and P95 also showed the highest inhibitory effect on PGE_2_ but less than SO with IC_50_ as 13.72 ± 0.81 *μ*g/ml, 12.26 ± 0.71 *μ*g/ml, and 8.61 ± 2.23 *μ*g/ml, respectively. 2,3-Pinanediol was the major anti-inflammatory compound analyzed in PH (11.28%) and P95 (19.82%) while terpinen-4-ol constituted a major anti-inflammatory compound in SO at 35.13%. These findings are the first supportive data for ethnomedical use for analgesic and anti-inflammatory activity in acute (SO) and chronic (PH and P95) inflammation.

## 1. Introduction

Pain is a common symptom and sign of inflammation and tissue damage [1–3]. Etiology including physical, biological, and chemical factors such as trauma, overuse, chemical, toxins, and pathogens can activate inflammatory response [1]. Inflammation is a response to protect and restore cells and tissues to a normal state [4]. The stimulus activates leukocytes to produce inflammatory cytokines such as tumor necrosis factor-*α* (TNF-*α*) [1]. In a site of tissue injury, prostaglandin *E*_2_ (PGE_2_) plays an important role in acute inflammation and causes vasodilation edema, acute pain, and fever [5]. TNF-*α* is an inflammatory cytokine that is intertwined with PGE_2_ as it stimulates phospholipase *A*_2_ and releases eicosanoids from the cyclooxygenase and lipoxygenase pathways in arachidonic acid metabolism [5]. The important product from cyclooxygenase is PGE_2_ [5]. Additionally, high levels of TNF-*α* can trigger fever and activate endothelial cells to express adhesion molecules resulting in leukocytes adherence and prolonged inflammation [6]. Macrophages trigger production of TNF-*α* cytokines causing pain and fever, loss of cell function, or loss of mobility in joints [2]. TNF-*α* can also activate macrophages to produce nitric oxide (NO) [7]. NO is a free radical derived from L-arginine and oxygen by inducible nitric oxide synthase (iNOS) enzyme from macrophages [8]. NO induces toxicity by interaction with superoxide and produces peroxynitrite which is highly toxic to microorganisms and normal neighboring cells [8]. Cells and tissues are gradually destroyed by excessive NO production, and as a result, the perception of pain remains.

Although the outcome of inflammatory responses involves physiological functions to protect and restore cells and tissues to a normal state, excessive inflammatory response is the cause of persistent inflammation and leads to chronic inflammation and pain [9, 10]. The impact of chronic inflammation involvement in chronic diseases such as systemic lupus erythematosus (SLE), rheumatoid arthritis, osteoarthritis, cancer, and cardiovascular diseases is well known [10].

Herbal remedies have been used for their anti-inflammatory and pain-relieving properties according to folk wisdom and in traditional ethnomedicine for centuries. According to Thai traditional medicine principles, herbs which have a spicy taste and pungent aroma such as capsicum, ginger, and plai (*Zingiber cassumunar*) are often used for pain relief [11].

The chemotaxonomy study of some *Zanthoxylum* species such as *Z. acanthopodium*, *Z. nitidum*, and *Z. myriacanthum* are found in Northern Thailand [12, 13] or *Z. budrunga*, *Z. bungeanum*, and *Z. schinifolium*, all have shown anti-inflammatory and antinociceptive action [14–17]. *Zanthoxylum rhetsa* is a pungent plant and a member of the Rutaceae family. Its whole fruit consists of pericarp and seed and is used in the diet in the Northern part of Thailand. Both pericarp and fruit are described in Pra-O-Sod-Pra-Narai scripture and Thai Traditional Household Remedy for muscle spasm, a pain relief from swelling of muscle and tendons and also as pain relief from abscesses and hemorrhoids [11, 18]. *Z. rhetsa* fruit is also extensively used as an anti-inflammatory agent and antiseptic in India [19]. *Z. rhetsa* fruit and seed are also used as a pain relief treatment from toothache, digestion problems, inflammation, and infection in Southeast Asia [19]. *Z. rhetsa* activity is a mosquito repellent, and its larvicidal, antimicrobial, antioxidant, and antitumor activities have been characterized [20]. Additionally, major chemical compounds in pericarp, fruit, and seed of *Z. rhetsa* as monoterpenes such as limonene, terpinen-4-ol, sabinene, and *α*-pinene [21–30] have been reported for their anti-inflammatory activity [31–33].

Therefore, the present study compared and investigated the anti-inflammatory activity of various anatomical parts such as whole fruits, pericarp, and seed of *Z. rhetsa* extracts through the inhibition of lipopolysaccharide- (LPS-) induced NO, TNF-*α*, and PGE_2_ in RAW264.7 macrophages. Additionally, chemical compositions of the active extracts were also delineated as anti-inflammatory, and pain relief activity of *Z. rhetsa* has been poorly studied [21–23]. Furthermore, the analysis of chemical constituents in pericarp, fruit, and seed of *Z. rhetsa* of various extractions and characterizing the anti-inflammatory activity has not been undertaken [31–33].

## 2. Materials and Methods

### 2.1. Plant Materials

*Z. rhetsa* was collected from its natural habitat in Ban Mae Khaw Tom Thasud village, Muang district, Chiang Rai province, Thailand. The voucher specimen was identified by using important characteristic of the morphology of both flower and fruit. After that, the scientific name of plant material was identified by botanists in the Department of National Parks, Wildlife and Plant Conservation, Bangkok, Thailand. The voucher specimen BKF number 193835 was preserved in the office of the Forest Herbarium, Bangkok, Thailand.

### 2.2. Chemicals and Reagents

Ethanol 95% (EtOH) (commercial grade) was purchased from C.M.J. Anchor Company (Thailand). Analytical grade dimethyl sulfoxide (DMSO), hexane, hydrochloric acid (HCl), and isopropanol were purchased from RCI Labscan (Thailand). Distilled water was produced by Milli-Q water purification system from Millipore (USA). Griess reagent (1% sulfanilamide and 0.1% *N*-(1-naphthyl) ethylenediamine dihydrochloride in 2.5% phosphoric acid), thiazolyl blue tetrazolium bromide (MTT), lipopolysaccharide (LPS) from *E.coli* (O55:B5), and prednisolone were purchased from Sigma-Aldrich (USA). Fetal bovine serum (FBS), penicillin-streptomycin (P/S), RPMI 1640 medium, and Dulbecco's modified eagle medium (DMEM) were purchased from Gibco (USA). The prostaglandin *E*_2_ ELISA kit was purchased from Cayman Chemical (USA), and Mouse TNF-*α* Quantikine ELISA test kit was purchased from R&D System Inc (USA).

### 2.3. Preparation of Extracts

After plant materials were sun-dried, they were separated into pericarp, fruit, and seed. Each part was ground to coarse powder and then was extracted by 3 methods consisting of maceration with hexane, 95% EtOH and 50% EtOH, water distillation, and decoction.

For maceration: each part powder (1 kg) was extracted by maceration with different solvent for three days (solvent: powder ratio = 2 : 1) and filtered through Whatman no.1 filter paper. The marc was remacerated twice, and the combined filtrate was evaporated by rotary evaporator to give the hexane extract, 95% ethanolic extract, and 50% ethanolic extract of pericarp (PH, P95, and P50), fruit (FH, F95, and F50), and seed (SH, S95, and S50), respectively.

For water distillation: each part powder (500 g) was distilled in a Clevenger apparatus for 100 minutes and the essential oil was collected and gave the essential oil from pericarp (PO), fruit (FO), and seed (SO).

**Figure 1:**
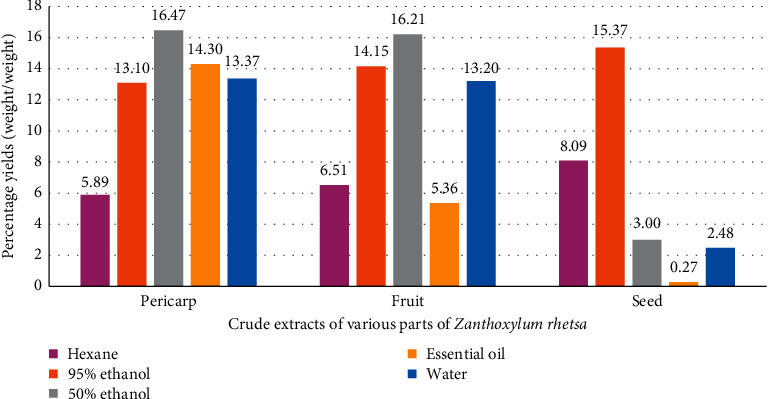
The percentage yields of the crude extracts of various parts of *Z. rhetsa*.

For decoction: each part of powder (500 g) was boiled in distilled water for 15 minutes and filtered. The residue had twice repeated decoction, and the combined filtrate was reduced to 1/3 by boiling then freeze dried to give the water extract from pericarp (PW), fruit (FW), and seed (SW). All crude extracts showed percentage of yield on Figure 1. The crude extracts were kept at −20°C, and the essential oils were kept at 4°C before use.

### 2.4. Cell Culture and Culture Media

RAW 264.7 macrophages from mouse (*Mus musculus*) were purchased from American Type Culture Collection (ATCC®TIB-71) (USA). Cells were cultured in two types of media according to assays: (1) RPMI 1640 medium for the assays of inhibition of LPS-induced nitric oxide (NO) and tumor necrosis factor-*α* (TNF-*α*) production following the established method [34] and the procedure in the manufacturer's manual [35], respectively, and (2) DMEM medium for the assay of inhibition of LPS-induced prostaglandin *E*_2_ (PGE_2_) production following the method of the procedure in the manufacturer's manual [36]. Each medium was supplemented with 10% FBS and 1% P/S (100 unit/ml) and incubated in an incubator at 37°C, 5% CO_2_, and 95% humidity.

### 2.5. Determination of Cell Viability

Cell viability was done in triplicate by using MTT assay [34]. Briefly, after 1 × 10^5^ cells/well of RAW 264.7 macrophages were seeded in sterilized 96 well-plate (100 *μ*l/well) and incubated for 24 h, the medium was removed and replaced with 100 *μ*l/well of fresh medium. Various dilutions of samples were added (100 *μ*l/well) and incubated for another 24 h. Subsequently, the supernatants (100 *μ*l/well) were removed, and the viable cells were determined by adding 10 *μ*l/well of the MTT solution (5 mg/ml) and further incubated for 2 h. The medium was then removed and replaced with 100 *μ*l/well of isopropanol containing 0.04 M HCl to dissolve formazan in the cells. The absorbance was measured by microplate reader at 570 nm. Cell viability that was higher than 70% compared with control (control medium for water extracts and control solvent: 0.2% DMSO of final concentration for crude extracts, essential oils, and prednisolone) indicated that the activity of the tested samples was not due to cytotoxicity [34]. The percentage of cell viability was calculated by using the following equation:

(1) $$\begin{array}{c} \text{\% cell viability}=\left[\frac{\text{OD sample}}{\text{OD control}}\right]\;\times 100, \end{array}$$

where OD = optical density; OD sample = mean of sample ODs; OD control = mean of control ODs.

### 2.6. Anti-Inflammatory Activities

#### 2.6.1. Determination of Inhibition of LPS-Induced NO Production

The determination of inhibitory effect of LPS-induced NO production was done in triplicate following the protocol of an established method [34]. Briefly, 100 *μ*l/well of RAW 264.7 macrophages (1 × 10^5^ cells/well) were seeded in sterilized 96 well-plate and incubated for 24 h, and then the medium was removed and replaced with 100 *μ*l/well of fresh medium containing LPS (2 ng/ml of final concentration). Various dilutions of samples were added (100 *μ*l/well) and incubated for another 24 h. Subsequently, a 100 *μ*l/well of supernatant was transferred into a nonsterilized 96 well-plate and added with Griess reagent (100 *μ*l/well). The absorbance of the mixed solution was measured by microplate reader at 570 nm. The result of the tested sample was compared with that of prednisolone, a positive control. The percentage of the inhibition of LPS-induced NO production was calculated by using the following equation, and IC_50_ values were calculated by using GraphPad Prism software (CA, USA):

(2) $$\begin{array}{c} \text{\% inhibition}=\left[\frac{\text{OD control}-\text{OD sample}}{\text{OD control}}\right]\;\times 100, \end{array}$$

where OD = optical density; OD control = mean of control ODs (+LPS) – mean of control ODs (−LPS); OD sample = mean of sample ODs (+LPS) – mean of sample ODs (−LPS).

#### 2.6.2. Determination of Inhibition of LPS-Induced TNF-*α* Production

The inhibition of LPS-induced TNF-*α* production was determined by using Mouse TNF-*α* Quantikine ELISA test kit following the procedure in the manufacturer's manual [35]. Briefly, RAW 264.7 macrophages (1 × 10^5^ cells/well) were seeded in sterilized 96 well-plate (100 *μ*l/well) and incubated for 24 h; then, the medium was removed and replaced with 100 *μ*l/well of fresh medium containing LPS at 2 ng/ml final concentration. Various dilutions of samples were added (100 *μ*l/well) and incubated for another 24 h. After incubation, the supernatant (50 *μ*l/well) was transferred into 96 well-plate of ELISA kit and it was carried out according to the method in the manufacturer's manual [35]. The absorbance was measured at 450 nm by using the microplate reader. The result of the tested sample was compared with that of prednisolone, a positive control. The experiment was conducted in triplicate. The percentage of the inhibition of LPS-induced TNF-*α* production was calculated by using the following equation, and IC_50_ values were calculated by using GraphPad Prism software (CA, USA):

(3) $$\begin{array}{c} \text{ \% inhibition}=\left[\frac{\text{OD control}-\text{OD sample}}{\text{OD control}}\right]\;\times 100, \end{array}$$

where OD = optical density; OD control = mean of control ODs (+LPS) – mean of control ODs (-LPS); OD sample = mean of sample ODs (+LPS) – mean of sample ODs (−LPS).

#### 2.6.3. Determination of Inhibition of LPS-Induced PGE_2_ Production

The inhibition of LPS-induced PGE_2_ production was determined by using prostaglandin *E*_2_ ELISA Kit-Monoclonal following the procedure in the manufacturer's manual [36]. Briefly, RAW 264.7 macrophages (1 × 10^5^ cells/well) were seeded in sterilized 96 well-plate (100 *μ*l/well) and incubated for 24 h, and then the medium was removed and replaced with 100 *μ*l/well of fresh medium containing LPS at 5 *μ*g/ml final concentration. Various dilutions of samples were added (100 *μ*l/well) and incubated for another 24 h. After incubation, the supernatant (50 *μ*l/well) was transferred into 96 well-plate of ELISA kit and the procedure carried out according to the method in the manufacturer's manual [36]. The absorbance was measured at 412 nm by using the microplate reader. The result of tested sample was compared with that of prednisolone, a positive control. The experiment was conducted in triplicate. The percentage of the inhibition of LPS-induced PGE_2_ production was calculated by using the following equation, and IC_50_ values were calculated by using GraphPad Prism software (CA, USA):

(4) $$\begin{array}{c} \text{\% inhibition }=\left[\frac{\text{mean OD sample }\left(+\text{LPS}\right)-\text{mean OD control }\left(+\text{LPS}\right)\;}{\text{mean OD control }\left(-\text{LPS}\right)-\text{mean OD control }\left(+\text{LPS}\right)}\right]\;\times 100, \end{array}$$

where OD = optical density.

#### 2.6.4. Chemical Composition Analysis by Gas Chromatography/Mass Spectrometry (GC/MS)

The chemical compositions of the active extracts were analyzed by using a Thermo Focus GC, Polaris *Q* with an autoinjector and a capillary column TG-5 slims (30 m × 0.25 mm × 0.25 *μ*m) (Thermo Fisher Scientific). Column oven temperature was programmed using the initial temperature at 60°C and 5 min initial time and then heated at the rate of 5°C/min to 300°C and held for 5 min. The injector temperature was 200°C, helium (He) was used as the carrier gas with constant flow rate of 1.0 ml/min, and the injection volume was 2 *μ*l (splitting ratio 1 : 50). The ionization energy was 70 eV. Mass spectrum of the GC/MS peak was detected by mass spectrometry and compared with library database of the National Institute of Standards and Technology (NIST 08, MD, USA) which matches the score for all compounds analyzed more than 870 would be selected [37]. Chemical composition analysis was carried out by the Herb and Thai Traditional Medicine Division, Thailand Science Park.

#### 2.6.5. Statistical Analysis

Cell viability, percentage of the inhibition of LPS-induced NO, TNF-*α* and PGE_2_ production, and IC_50_ were presented as mean ± standard error of means (SEM). Comparison of means between control and treatment groups was done by one-way analysis of variance followed by Dunnett's multiple comparison test. Comparison of means in between independent treatment groups (2 groups) was analyzed by using unpaired *t* test. Comparison of means in multiple treatment groups (≥3 groups) was analyzed by using one-way analysis of variance followed by one-way ANOVA. The level of significant difference was *p* < 0.05.

## 3. Results

### 3.1. Preparation of Extract

The percentage yields of extracts and essential oils are shown in Figure 1. The pericarp showed the highest yield of extraction by three methods such as 50% ethanol, oil part, and water extract (16.47%, 14.30%, and 13.37%, respectively). The seed showed the highest yield of extraction by hexane and 95% ethanol.

### 3.2. Determination of Cell Viability

**Figure 2:**
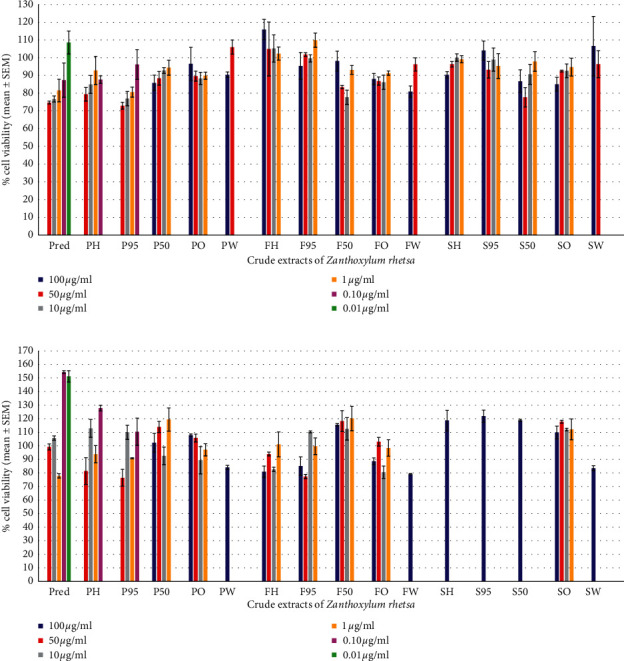
Cell viability of the various extracts of *Z. rhetsa* and prednisolone (Pred) at various concentrations (*n* = 3). (a) Viable cells for inhibition of LPS-induced NO and TNF-*α* production and (b) viable cells for inhibition of LPS-induced PGE_2_ production.

Cell viability after exposure to the various extracts of *Z. rhetsa* and prednisolone (Pred) (positive control) is presented in Figure 2(a) for inhibition of LPS-induced NO and TNF-*α* production and in Figure 2(b) for inhibition of LPS-induced PGE_2_ production. The various extracts of *Z. rhetsa* and prednisolone (Pred) showed greater than 70% cell viability at all concentrations when they were tested.

### 3.3. Determination of Inhibition of LPS-Induced NO Production

Anti-inflammatory activity of the various extracts of *Z. rhetsa* via the inhibition of NO production by the induction of LPS in RAW 264.7 macrophages compared with prednisolone (positive control) is shown in Table 1.

**Figure 3:**
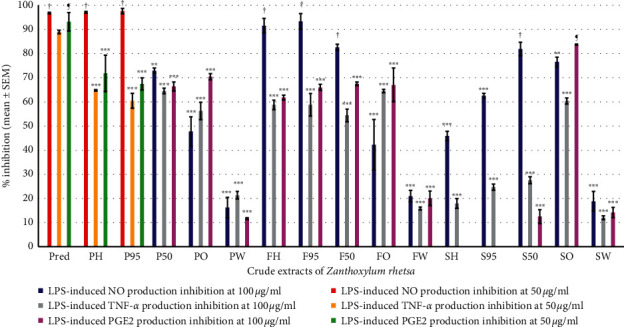
Percentage of the inhibition on LPS-induced NO, TNF-*α* and PGE_2_ production in RAW264.7 macrophages of crude extracts of *Z. rhetsa* and prednisolone at 100 *μ*g/ml and 50 *μ*g/ml (*n* = 3). † and ¶: the %inhibition on LPS-induced NO and PGE_2_ production, respectively, which were not different significantly from prednisolone. ^*∗∗*^*p* value < 0.01, ^*∗∗∗*^*p* value < 0.001 compared with prednisolone.

PH and P95 at 50 *μ*g/ml gave the highest %inhibition of NO production (97.15% ± 0.37 and 97.66% ± 1.12, respectively) while FH, F95, F50, and S50 at 100 *μ*g/ml gave the highest %inhibition of NO production (91.55% ± 3.04, 93.36% ± 3.23, 82.62% ± 1.26, and 81.94% ± 2.79, respectively). These results were not significantly different from prednisolone at 50 *μ*g/ml (96.82% ± 0.34) (Figure 3).

The extract results showed that PH and P95 had an inhibitory effect on NO production with IC_50_ values as 11.99 ± 1.66 *μ*g/ml and 15.33 ± 1.05 *μ*g/ml, respectively. They were significantly different (*p* value < 0.01 and *p* value < 0.001, respectively) from prednisolone (IC_50_ = 0.07 ± 0.001 *μ*g/ml or 0.19 ± 0.001 *μ*M). However, the pericarp was macerated in hexane and 95% ethanol. The results demonstrated with the whole fruits macerated in hexane and 95% ethanol showed higher activity than decoction in water and maceration in 50% ethanol. For seeds which underwent water distillation, significant anti-inflamatory activity on NO production was demonstrated compared to other extraction means. The method of extraction revealed the most activity in the pericarp on the inhibition of NO production which was demonstrated with maceration in 95% ethanol and hexane. All water extracts (PW, FW, and SW), the essential oil of both percarp (PO) and fruits (FO), and the hexane extract of seed (SH) were not active (IC_50_ > 100 *μ*g/ml).

### 3.4. Determination of Inhibition of LPS-Induced TNF-*α* Production

PH and P95 at 50 *μ*g/ml gave the highest %inhibition of TNF-*α* production (64.79% ± 0.26 and 60.46% ± 3.07, respectively) which were significantly different (*p*-value < 0.001) from prednisolone at 50 *μ*g/ml (89.00% ± 0.70) as the same as other extracts at 100 *μ*g/ml which gave the highest %inhibition of TNF-*α* production which were significantly different (*p* value < 0.001) from prednisolone at 50 *μ*g/ml (Figure 3).

The results of IC_50_ on inhibitory effect of TNF-*α* production are shown in Table 2. The pericarp which was macerated in hexane and 95% ethanol maintained inhibitory effects of NO production. PH and P95 were 36.08 ± 0.55 *μ*g/ml and 34.90 ± 2.58 *μ*g/ml, respectively, but were significantly different (*p* value < 0.001) from prednisolone (IC_50_ = 0.08 ± 0.003 *μ*g/ml or 0.22 ± 0.003 *μ*M). The IC_50_ of SO (49.85 ± 4.29 *μ*g/ml) was significantly different (*p* value < 0.05) from PH and P95. All water extracts (PW, FW and SW) and all extracts of the seed (except for the essential oil of the seed: SO) did not have the activity on LPS-induced TNF-*α* production inhibition (IC_50_ > 100 *μ*g/ml).

### 3.5. Determination of Inhibition of LPS-Induced PGE_2_ Production

SO at 100 *μ*g/ml gave the highest %inhibition of PGE_2_ production (83.70% ± 0.22) which were not significantly different from prednisolone at 50 *μ*g/ml (93.20% ± 3.80), while PH and P95 at 50 *μ*g/ml gave the highest %inhibition of PGE_2_ production (71.83% ± 7.51 and 67.44% ± 2.53, respectively) which were significantly different (*p* value < 0.001) from prednisolone (Figure 3).

The results on inhibitory effect on PGE_2_ production are shown in IC_50_ values (Table 3); SO exhibited the highest anti-inflammatory effect on PGE_2_ with IC_50_ as 8.61 ± 2.23 *μ*g/ml and was significantly different (*p* value < 0.05) from prednisolone (IC_50_ = 0.07 ± 0.003 *μ*g/ml or 0.19 ± 0.003 *μ*M). The inhibitory effect on PGE2 production of PH and P95 (IC50 = 13.72 ± 0.8 and 12.26 ± 0.71 *μ*g/ml) were not significantly different with SO but they were significantly different with prednisolone. However, its pericarp demonstrated higher anti-inflammatory activity on the inhibitory effect of PGE_2_ production than whole fruit and seed accept only seed oil (SO). All water extracts (PW, FW, and SW) and all extracts of the seed (except the essential oil of the seed: SO) did not have the activity on LPS-induced TNF-*α* production inhibition (IC_50_ > 100 *μ*g/ml).

### 3.6. Chemical Composition Analysis by Gas Chromatography/Mass Spectrometry (GC/MS)

**Figure 4:**
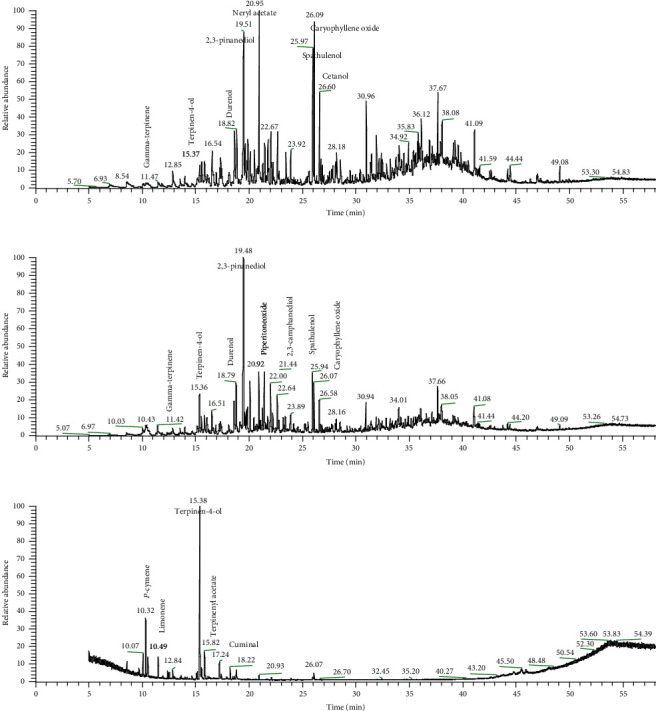
GC/MS chromatogram of active extracts of *Z. rhetsa*. (a) The hexane extract from pericarp (PH), (b) the 95% ethanolic extract from pericarp (P95), and (c) the essential oil from seed (SO).

PH and P95 showed the highest production inhibition of LPS-induced NO, TNF-*α*, and PGE_2_ while SO showed the highest production inhibition of LPS-induced PGE_2_. Therefore, PH, P95, and SO compositions were analyzed by GC/MS (Table 4) and presented GC/MS chromatogram of PH (Figure 4(a)), P95 (Figure 4(b)), and SO (Figure 4(c)). PH and P95 contained some chemical compounds as in SO; these were *γ*-terpinene (0.68%, 0.79%, and 4.91%, respectively), terpinen-4-ol (1.07%, 3.38%, and 35.13%, respectively), and terpinenyl acetate (1.57%, 1.62%, and 6.65%, respectively). PH and P95 shared similar composition but different in percentages. Bicyclo(3.1.1)heptane-2,3-diol,2,6,6-trimethyl or 2,3-pinanediol (11.28%), neryl acetate (7.65%), caryophyllene oxide (7.50%), spathulenol (6.65%), and cetanol (3.78%) are constituents in top 5 of PH. Bicyclo(3.1.1)heptane-2,3-diol,2,6,6-trimethyl or 2,3-pinanediol (19.82%), 2,3-camphanediol (5.87%), durenol (4.53%), piperitone oxide (4.46%), and spathulenol (4.39%) are in top 5 constituents of P95. Terpinen-4-ol was the major compound (35.13%) in SO; the next top 5 compounds were *p*-cymene (10.95%), terpinenyl acetate (6.65%), cuminol (5.60%), and limonene (5.48%).

## 4. Discussion

Pain may be acute or chronic depending on the duration of inflammatory response in the body [38, 39]. Inflammatory mechanisms assist in eliminating pathogens or stimulating wound healing in order to protect and restore cells and tissues into normal physiological functions [4]. Inflammatory responses, resulting in excessive release of inflammatory mediators and cytokines, can lead to tissue damage, chronic disease, and pain [9, 10]. Although medication can be effective for pain relief from inflammation, side effects from medication (i.e., steroid, NSAIDs, opioids, acetaminophen, etc) are significant. Herbal medicine is considered and utilized as a natural alternative for treatment of pain relief with potential to avoid some side-effects [40].

After cell and tissue damage, the body perceives pain. An acute inflammatory mechanism is induced by inflammatory mediators. PGE_2_ is the one of chemical mediators: histamine, substance P, bradykinin, acetylcholine, leukotrienes, and prostaglandins, resulting in heat, redness, swelling, and nociception. PGE_2_-induced vasodilation in the first step of acute inflammatory mechanism leads to increase microvascular permeability and induces pain by acting on peripheral sensory neurons [41]. The inhibition of PGE_2_ production can be effective to reduce heat, redness, edema, and pain. In the present study, PH, P95, and SO of *Z. rhetsa* were the most potent groups (IC_50_ < 20 *μ*g/ml) which showed the greatest potency of LPS-induced PGE_2_ production in RAW264.7 macrophages, while PO was the second most potent group (IC_50_ < 30 *μ*g/ml); P50, FO, and F95 was in the third group for potency (IC_50_ < 50 *μ*g/ml), and other extracts of *Z. rhetsa* were weak to inactive (IC_50_ > 50 *μ*g/ml). These results indicate that whole *Z. rhetsa* fruit should be separated into pericarp and seed, and the inhibitory effect of PGE_2_ production is higher as a consequence. A previous study reported that an ethanolic extract from *Z. rhetsa* fruit (consisting of pericarp and seed) could inhibit COX-1 (90.80%) and COX-2 (94.40%) [21]. PGE_2_ is one of the products derived from cyclooxygenase pathway [5]; therefore, PH, P95, and SO may reduce acute pain from an acute inflammatory mechanism through inhibition of COX-1 and COX-2 as well as the eicosanoid product PGE_2_. Additionally, an *in vivo* study on a bioadhesive gel containing essential oil from the fruit could inhibit licking behavior, edema, and redness of the buccal cavity in rats [22] which was also due to reduced PGE_2_ in acute inflammation. In clinical trials, a massage oil containing essential oil from fruit relieved pain in the calf muscle compared with carrier oil (placebo) in healthy volunteers after induction by standing and heel raise [23].

Though the previous study was done on whole fruit, our study has shown that *Z. rhetsa* pericarp and seed could perform the same pharmacological functions. This is an important finding since it would be the preparation of this herbal medicine from pericarp or seed not only whole fruit. Although the percentage yields of SO was less (0.27%) (Figure 1), the preparation of the distillation of the seed should be studied further in order to increase its yield. The present study also showed the highest %inhibition of PGE_2_ production of SO at 100 *μ*g/ml (83.70% ± 0.22) which was not significantly different from prednisolone at 50 *μ*g/ml (93.20% ± 3.80) while PH and P95 at 50 *μ*g/ml showed the highest %inhibition of PGE_2_ production (71.83% ± 7.51 and 67.44% ± 2.53, respectively) which was significantly different (*p* value < 0.001) from prednisolone (Figure 3), whereas IC_50_ values of PH and P95 were not significantly different from SO (Table 3). Our result was indicated; the preparation of analgesic and anti-inflammatory agents in acute inflammation from PH, P95, and SO was apparent. Whereas percentage yields of PH (5.89%) and P95 (13.10%) were higher than SO (0.27%) (Figure 1). Our study is also the first report on anti-inflammatory activity of PH, P95, and SO from *Z. rhetsa* by the inhibition of PGE_2_ production in RAW 264.7 macrophages.

TNF-*α* is an inflammatory cytokine which releases in both acute and chronic inflammation; TNF-*α* induces pain and fever and plays a role in rheumatoid arthritis, osteoarthritis, and systemic lupus erythematosus [42]. Thai ethnomedicine use of *Z. rhetsa* was able to demonstrate the anti-inflammatory action in joints. *Z. rhetsa* fruit is used as an oil (named Pa-Ra-Ti-Tri) and ointment (named Bee-Pra-Sen) for treatment of muscle and joint inflammation in Thai ancient scripture (named Pra-O-Sod-Pra-Na-Rai) [18]. Additionally, our extracts were effective on TNF-*α* production by PH and P95, whereas SO was less active. Therefore, PH and P95 may relieve pain and inflammation via inhibition of TNF-*α* production. Our findings could be utilized to improve ethnomedicine use by developing a topical analgesic remedy from PH or P95 which demonstrates clinical utility.

PH and P95 also demonstrated the highest potency in the inhibition of NO production, which is a free radical synthesized by inducible nitric oxide synthase (iNOS) from macrophages with L-arginine as a precursor [8]. Increasing concentrations of nitrite in synovial fluid of joints are related to rheumatoid arthritis and osteoarthritis [43]. Therefore, PH and P95 could protect cells and tissues from injury due to NO. Our results also demonstrated the highest potency on the % inhibition of NO production by PH (97.15% ± 0.37) and P95 (97.66% ± 1.12) at 50 *μ*g/ml which were not significantly different from prednisolone (96.82% ± 0.34) at 50 *μ*g/ml (Figure 3); therefore, PH and P95 may relieve pain from inflammation. FH and F95 at 100 *μ*g/ml gave the highest %inhibition of NO production (91.55% ± 3.04 and 93.36% ± 3.23, respectively) which were not significantly different from prednisolone (96.82% ± 0.34) at 50 *μ*g/ml (Figure 3). These results indicate the potency of *Z. rhetsa* pericarp is higher than *Z. rhetsa* fruit for use as anti-inflammatory agent due to infection. Ethnomedicine use of *Z. rhetsa* fruit was able to demonstrate the anti-inflammatory action due to infection by a component in the Ma-Ha-Wat-Ta-Na remedy for the treatment of abscesses in Pra-O-Sod-Pra-Na-Rai ancient scripture [18].

Additionally, both NO and TNF-*α* have important roles in progressive osteoarthritis and rheumatoid arthritis [44–46]. TNF-*α* stimulates chondrocytes in cartilages to produce high levels of NO [44]. PH and P95 may reduce pain, swelling, and tissue damage through inhibiting NO and TNF-*α* production.

All extracts and essential oils from *Z. rhetsa* and prednisolone (positive control) showed greater than 70% cell viability at all concentrations (Figure 2) when tested, indicating that compounds were not cytotoxic to the cells, and their anti-inflammatory activity via the inhibition of LPS-induced NO, TNF-*α*, and PGE_2_ production in RAW 264.7 macrophages was not due to cytotoxicity [34].

Additionally, our extraction methods and results were supportive data for the Thai traditional preparation of drugs as the extraction by hexane is similar to preparation of the folk method called Hung-Nam-Mun (hot oil extract) [47]. These methods extensively use coconut oil for frying plant materials; however, a rancid odor because of coconut oil is apparent. Whereas maceration in hexane has no odor and a high extraction yield.

Some compounds analyzed in the active extracts, PH, P95, and SO (Table 4), had previously been reported to inhibit inflammatory mediators. Terpinen-4-ol was found in both PH (1.07%), P95 (3.38%), and SO (35.13%), and previous studies reported that terpinen-4-ol could inhibit TNF-*α*, IL-1*β*, and PGE_2_ production by LPS-activated human blood monocytes [31]. The second most abundant compounds in SO, *p*-cymene (10.95%), has previously been demonstrated to exhibit analgesic and anti-inflammatory properties in mice [48, 49]. Cuminaldehyde (5.60%) competitively inhibited the activity of 15-lipoxygenase, an enzyme involved in the production of inflammatory mediators such as leukotrienes, using lipoxygenase inhibition assay [50]. Limonene (5.48%) was found to be in the top five compounds of SO and also previously shown to suppress the production of LPS-induced NO, PGE_2_, TNF-*α*, IL-1*β*, and IL-6 [33]. The occurrence of these compounds in SO was the reasons for its in vitro activities. The compounds in PH and P95, spathulenol (6.65% and 4.39%, respectively) and caryophyllene oxide (7.50% and 3.66%, respectively), previously showed that they could inhibit the production of NO, IL-1*β*, and IL-6 [32]. The major component in PH and P95 was found to be bicyclo(3.1.1)heptane-2,3-diol, 2,6,6-trimethyl or 2,3-pinanediol (11.28% and 19.82%). This compound was earlier reported as an agent that increased microcirculation when applied topically [51]; thus, 2,3-pinanediol could contribute to pain relief when applied in PH and P95 on inflamed areas [52].

## 5. Conclusions

PH, P95, and SO of *Z. rhetsa* exerted pain-relieving and anti-inflammatory activity through inhibition of inflammatory mediators via LPS-induced NO, TNF-*α*, and PGE_2_ in RAW264.7 macrophages. Our study suggests that the PH and P95 extract fractions analyzed could provide constituents suitable for pain relief in chronic inflammation due to their activity on NO and TNF-*α* and SO inhibitory effect on PGE_2_ production. Moreover, PH, P95, and SO contained terpinen-4-ol that was previously reported as an inhibitor of LPS-induced PGE_2_ and TNF-*α*. Other components in SO, *p*-cymene, and limonene have previously been reported for their in vitro and in vivo anti-inflammatory activity. Therefore, SO may have potential for the development into an analgesic and anti-inflammatory product for inflammation, and its active constituents should be further refine or studied further with additional reference standards where possible. A main active constituent determined in PH and P95 which enables inhibition of NO, TNF-*α*, and PGE_2_ appears to be 2,3-pinanediol which comprises almost 20% of P95. These findings are the first foundational supportive data for ethnomedical use as anti-inflammatory and analgesic herbal medicine treatment. *Z. rhetsa* pericarp that is macerated with hexane and 95% ethanol and seed essential oil are now being studied for analgesic product development in ongoing studies in our laboratories.

## Figures and Tables

**Table 1:** Inhibitory effect and IC50 values of LPS-induced nitric oxide (NO) production in RAW264.7 macrophages of various *Zanthoxylum rhetsa* extracts.

Part of plant	Extract and positive control	Code	Percentage of inhibition at various concentrations						IC_50_ (*μ*g/ml)
			100 *μ*g/ml	50 *μ*g/ml	10 *μ*g/ml	1 *μ*g/ml	0.10 *μ*g/ml	0.01 *μ*g/ml	
Pericarp	Hexane	PH	—	97.15 ± 0.37†	38.47 ± 8.69	−11.70 ± 3.94	−12.38 ± 3.61	—	11.99 ± 1.66^*∗∗*^^,a^
	95% ethanol	P95	—	97.66 ± 1.12†	24.46 ± 2.71	−15.60 ± 3.58	−15.65 ± 2.27	—	15.33 ± 1.05^*∗∗∗*^^,a^
	50% ethanol	P50	72.96 ± 1.04^*∗∗*^	35.78 ± 1.83	3.10 ± 2.50	−1.68 ± 4.25	—	—	67.55 ± 2.22^*∗∗∗*^
	Essential oil	PO	47.75 ± 6.07^*∗∗∗*^	15.29 ± 3.43	−14.02 ± 1.79	−15.51 ± 2.41	—	—	>100^*∗∗∗*^
	Water	PW	16.23 ± 4.25^*∗∗∗*^	8.75 ± 1.53	—	—	—	—	>100^*∗∗∗*^

Fruit	Hexane	FH	91.55 ± 3.04†	60.95 ± 0.84	8.69 ± 3.68	−5.20 ± 4.00	—	—	39.81 ± 0.53^*∗∗∗*^^,c^
	95% ethanol	F95	93.36 ± 3.23†	72.35 ± 4.53	11.88 ± 1.01	−14.05 ± 6.36	—	—	29.42 ± 3.05^*∗∗∗*^^,b^
	50% ethanol	F50	82.62 ± 1.26†	48.32 ± 0.51	4.02 ± 1.80	−2.95 ± 0.87	—	—	51.63 ± 0.43^*∗∗∗*^
	Essential oil	FO	42.23 ± 10.48^*∗∗∗*^	15.29 ± 7.11	−13.02 ± 3.56	−11.27 ± 3.42	—	—	>100^*∗∗∗*^
	Water	FW	20.97 ± 2.36^*∗∗∗*^	8.77 ± 1.86	—	—	—	—	>100^*∗∗∗*^

Seed	Hexane	SH	45.92 ± 1.91^*∗∗∗*^	22.75 ± 1.03	−1.17 ± 5.23	−11.93 ± 8.02	—	—	>100^*∗∗∗*^
	95% ethanol	S95	62.56 ± 0.98^*∗∗∗*^	35.63 ± 1.36	2.52 ± 7.24	−3.69 ± 8.17	—	—	73.10 ± 1.55^*∗∗∗*^
	50% ethanol	S50	81.94 ± 2.79†	46.88 ± 1.10	5.86 ± 3.64	−10.45 ± 2.58	—	—	54.36 ± 1.21^*∗∗∗*^
	Essential oil	SO	76.57 ± 1.91^*∗∗*^	35.40 ± 3.07	−6.95 ± 2.64	−13.79 ± 2.95	—	—	65.34 ± 3.18^*∗∗∗*^
	Water	SW	18.83 ± 4.06^*∗∗∗*^	10.88 ± 3.05	—	—	—	—	>100^*∗∗∗*^
	Prednisolone	Pred	—	96.82 ± 0.34†	89.32 ± 0.31	81.49 ± 6.98	72.90 ± 2.26	5.16 ± 1.25	0.07 ± 0.001 (0.19 ± 0.001 *μ*M)

The results are shown as mean ± standard error of mean (SEM) (*n* = 3). LPS: lipopolysaccharide; IC_50_: the half maximal inhibitory concentration. †: the %inhibition was not different significantly from prednisolone; ^*∗∗*^*p* value < 0.01, ^*∗∗∗*^*p* value < 0.001 compared with prednisolone as a positive control; ^a^not significantly different between PH and P95; ^b^significantly different (*p* value < 0.01) between PH, P95, and F95; ^c^significantly different (*p* value < 0.05) between F95 and FH; (–) not tested.

**Table 2:** Inhibitory effect and IC50 values of LPS-induced tumor necrosis factor-*α* (TNF-*α*) production in RAW264.7 macrophages of various *Zanthoxylum rhetsa* extracts.

Part of plant	Extract and positive control	Code	Percentage of inhibition at various concentrations						IC_50_ (*μ*g/ml)
			100 *μ*g/ml	50 *μ*g/ml	10 *μ*g/ml	1 *μ*g/ml	0.10 *μ*g/ml	0.01 *μ*g/ml	
Pericarp	Hexane	PH	—	64.79 ± 0.26^*∗∗∗*^	22.26 ± 1.19	4.24 ± 9.04	−34.86 ± 12.07	—	36.08 ± 0.55^*∗∗∗*^^,a^
	95% ethanol	P95	—	60.46 ± 3.07^*∗∗∗*^	30.49 ± 10.19	15.66 ± 9.88	−16.05 ± 6.31	—	34.90 ± 2.58^*∗∗∗*^^,a^
	50% ethanol	P50	64.53 ± 1.14^*∗∗∗*^	45.57 ± 2.06	26.86 ± 0.76	17.22 ± 1.13	—	—	63.15 ± 3.82^*∗∗∗*^
	Essential oil	PO	56.24 ± 3.61^*∗∗∗*^	30.24 ± 4.30	1.03 ± 0.48	−7.37 ± 7.59	—	—	85.05 ± 3.24^*∗∗∗*^
	Water	PW	21.33 ± 1.57^*∗∗∗*^	—	—	—	—	—	>100^*∗∗∗*^

Fruit	Hexane	FH	58.77 ± 2.00^*∗∗∗*^	26.36 ± 0.55	0.59 ± 4.06	−11.31 ± 8.34	—	—	88.11 ± 1.85^*∗∗∗*^
	95% ethanol	F95	58.81 ± 4.68^*∗∗∗*^	21.74 ± 7.55	−1.13 ± 15.71	−16.78 ± 3.36	—	—	91.12 ± 3.42^*∗∗∗*^
	50% ethanol	F50	54.42 ± 2.59^*∗∗∗*^	30.00 ± 4.20	16.52 ± 4.33	0.53 ± 3.97	—	—	93.54 ± 4.02^*∗∗∗*^
	Essential oil	FO	64.54 ± 0.70^*∗∗∗*^	43.48 ± 3.46	12.06 ± 2.08	−16.44 ± 1.98	—	—	73.22 ± 3.85^*∗∗∗*^
	Water	FW	15.88 ± 0.60^*∗∗∗*^	—	—	—	—	—	>100^*∗∗∗*^

Seed	Hexane	SH	17.92 ± 2.03^*∗∗∗*^	—	—	—	—	—	>100^*∗∗∗*^
	95% ethanol	S95	24.69 ± 1.25^*∗∗∗*^	—	—	—	—	—	>100^*∗∗∗*^
	50% ethanol	S50	27.53 ± 1.47^*∗∗∗*^	—	—	—	—	—	>100^*∗∗∗*^
	Essential oil	SO	60.41 ± 1.24^*∗∗∗*^	50.23 ± 1.13	29.93 ± 0.19	20.31 ± 0.80	—	—	49.85 ± 4.29^*∗∗∗*^^,b^
	Water	SW	12.02 ± 0.83^*∗∗∗*^	—	—	—	—	—	>100^*∗∗∗*^
	Prednisolone	Pred	—	89.00 ± 0.70	77.18 ± 0.69	71.01 ± 2.74	56.05 ± 0.08	28.59 ± 2.59	0.08 ± 0.003 (0.22 ± 0.003 *μ*M)

The results are shown as mean ± standard error of mean (SEM) (*n* = 3). LPS: lipopolysaccharide; IC_50_: the half maximal inhibitory concentration. ^*∗∗∗*^*p* value < 0.001 compared with prednisolone as a positive control; ^a^not different significantly statistic between PH and P95; ^b^different significantly statistic (*p* value < 0.05) between PH, P95, and SO; (–) not tested.

**Table 3:** Inhibitory effect and IC50 values of LPS-induced prostaglandin *E*2 (PGE2) production in RAW264.7 macrophages of various *Zanthoxylum rhetsa* extracts.

Part of plant	Extract and positive control	Code	Percentage of inhibition at various concentrations						IC_50_ (*μ*g/ml)
			100 *μ*g/ml	50 *μ*g/ml	10 *μ*g/ml	1 *μ*g/ml	0.10 *μ*g/ml	0.01 *μ*g/ml	
Pericarp	Hexane	PH	—	71.83 ± 7.51^*∗∗∗*^	36.28 ± 1.19	3.28 ± 0.52	2.27 ± 0.07	—	13.72 ± 0.81^*∗∗*^^,a^
	95% ethanol	P95	—	67.44 ± 2.53^*∗∗∗*^	40.34 ± 1.77	2.79 ± 1.62	1.66 ± 1.37	—	12.26 ± 0.71^*∗∗*^^,a^
	50% ethanol	P50	66.39 ± 1.83^*∗∗∗*^	52.66 ± 1.82	36.74 ± 0.55	31.50 ± 3.95	—	—	42.30 ± 1.20^*∗∗∗*^^,c^
	Essential oil	PO	70.42 ± 1.26^*∗∗∗*^	63.32 ± 1.44	33.74 ± 0.62	18.84 ± 0.77	—	—	24.13 ± 2.03^*∗∗∗*^^,b^
	Water	PW	11.67 ± 0.39^*∗∗∗*^	—	—	—	—	—	>100^*∗∗∗*^

Fruit	Hexane	FH	61.81 ± 1.00^*∗∗∗*^	20.93 ± 2.47	6.24 ± 0.10	4.68 ± 0.002	—	—	87.15 ± 0.55^*∗∗∗*^
	95% ethanol	F95	66.00 ± 1.26^*∗∗∗*^	52.35 ± 0.28	39.33 ± 1.31	37.64 ± 1.55	—	—	43.24 ± 1.04^*∗∗∗*^^,c^
	50% ethanol	F50	67.51 ± 0.72^*∗∗∗*^	40.90 ± 2.76	22.51 ± 1.75	13.23 ± 0.95	—	—	69.97 ± 3.37^*∗∗∗*^
	Essential oil	FO	67.07 ± 6.94^*∗∗∗*^	54.22 ± 0.03	31.35 ± 4.12	26.08 ± 2.77	—	—	40.85 ± 1.99^*∗∗∗*^^,c^
	Water	FW	20.06 ± 3.00^*∗∗∗*^	—	—	—	—	—	>100^*∗∗∗*^

Seed	Hexane	SH	−9.09 ± 0.28^*∗∗∗*^	—	—	—	—	—	>100^*∗∗∗*^
	95% ethanol	S95	−15.24 ± 4.16^*∗∗∗*^	—	—	—	—	—	>100^*∗∗∗*^
	50% ethanol	S50	12.48 ± 2.82^*∗∗∗*^	—	—	—	—	—	>100^*∗∗∗*^
	Essential oil	SO	83.70 ± 0.22¶	69.15 ± 2.48	52.50 ± 4.25	31.86 ± 6.71	—	—	8.61 ± 2.23^*∗*^^,a^
	Water	SW	14.19 ± 2.12^*∗∗∗*^	—	—	—	—	—	>100^*∗∗∗*^
	Prednisolone	Pred	—	93.20 ± 3.80¶	87.74 ± 1.51	89.16 ± 1.82	81.57 ± 0.87	52.93 ± 1.35	0.07 ± 0.003 (0.19 ± 0.003 *μ*M)

The results are shown as mean ± standard error of mean (SEM) (*n* = 3). LPS: lipopolysaccharide; IC_50_: the half maximal inhibitory concentration. ¶the %inhibition was not different significantly from prednisolone; ^*∗*^*p* value < 0.05, ^*∗∗*^*p* value < 0.01, ^*∗∗∗*^*p* value < 0.001 compared with prednisolone as a positive control; ^a^not different significantly statistic between PH, P95, and SO; ^b^different significantly statistic (*p* value < 0.01) between PH, P95, and PO; ^c^not different significantly statistic between P50, F95, and FO; (–) not tested.

**Table 4:** Chemical profiles in the active extracts of *Zanthoxylum rhetsa* by GC/MS.

No.	Chemical composition	The active extracts								
		Hexane extract from pericarp (PH)			95% ethanolic extract from pericarp (P95)			Essential oil from seed (SO)		
		RT (min)	% Area	Match score	RT (min)	% Area	Match score	RT (min)	% Area	Match score
1	Sabinene	ND	ND	ND	ND	ND	ND	8.56	1.91	889
2	Alpha-phellandrene	ND	ND	ND	ND	ND	ND	9.69	1.49	887
3	Alpha-terpinene	ND	ND	ND	ND	ND	ND	10.07	4.07	879
4	*p*-Cymene	ND	ND	ND	ND	ND	ND	10.32	10.95	882
5	Limonene	ND	ND	ND	ND	ND	ND	10.49	5.48	884
6	Gamma-terpinene	11.47	0.68	885	11.42	0.79	885	11.47	4.91	878
7	Linalool oxide	ND	ND	ND	ND	ND	ND	11.92	0.57	880
8	Terpinolen	ND	ND	ND	ND	ND	ND	12.38	1.43	885
9	2-Methyl-1-phenylpropene	ND	ND	ND	ND	ND	ND	12.52	1.26	871
10	Linalool	ND	ND	ND	ND	ND	ND	12.84	1.63	877
11	Terpinen-4-ol	15.37	1.07	898	15.36	3.38	898	15.38	35.13	905
12	Terpinenyl acetate	15.81	1.57	892	15.80	1.62	892	15.82	6.65	901
13	L-carvone	17.24	1.07	874	17.22	1.01	874	ND	ND	ND
14	Cuminal	ND	ND	ND	ND	ND	ND	17.24	5.60	885
15	*p*-Cymen-3-ol	ND	ND	ND	ND	ND	ND	18.81	2.43	871
16	Durenol	18.82	3.71	880	18.79	4.53	880	ND	ND	ND
17	Bicyclo(3.1.1)heptane-2,3-Diol, 2,6,6-trimethyl	19.51	11.28	896	19.48	19.82	896	ND	ND	ND
18	Limonene oxide	20.11	2.07	873	20.09	4.03	873	ND	ND	ND
19	Nerol	ND	ND	ND	ND	ND	ND	20.93	0.87	878
20	Neryl acetate	20.95	7.65	872	20.92	4.28	872	ND	ND	ND
21	2,3-Camphanediol	21.46	2.57	880	21.44	5.87	880	ND	ND	ND
22	7-Tetradecene	21.76	1.81	873	21.73	0.91	873	ND	ND	ND
23	Linoleic acid	ND	ND	ND	ND	ND	ND	21.76	0.15	881
24	Piperitone oxide	22.04	3.16	872	22.00	4.46	872	ND	ND	ND
25	Lauric acid	25.60	0.87	879	25.53	1.01	879	ND	ND	ND
26	Spathulenol	25.97	6.65	890	25.94	4.39	890	ND	ND	ND
27	Caryophyllene oxide	26.09	7.50	889	26.07	3.66	889	ND	ND	ND
28	Cetanol	26.60	3.78	877	26.58	2.34	877	ND	ND	ND
29	Ethyl linoleolate	31.92	1.97	871	31.91	1.12	871	ND	ND	ND

GC/MS: gas chromatography/mass spectrometry; RT: retention time; min: minutes; ND: not detected.

## Data Availability

The data used to support the findings of this study are available within the article.
